# Skin Aging and Mitochondrial Dysfunction: Structural Changes, Mechanistic Insights, and Therapeutic Perspectives

**DOI:** 10.1155/omcl/5140711

**Published:** 2026-05-14

**Authors:** Nathália Cardoso de Afonso Bonotto, Elize Musachio, Giulliano Danezi Felin, Giancarllo Danezi Felin, Carla Helena Augustin Schwanke, Ivana Beatrice Mânica da Cruz, Fernanda Barbisan

**Affiliations:** ^1^ Postgraduate Program in Pharmacology, Universidade Federal de Santa Maria, Santa Maria, Rio Grande do Sul, Brazil, ufsm.br; ^2^ Postgraduate Program in Gerontology, Universidade Federal de Santa Maria, Santa Maria, Rio Grande do Sul, Brazil, ufsm.br; ^3^ Residency in General Surgery, University Hospital-Universidade Federal de Santa Maria, Santa Maria, Rio Grande do Sul, Brazil, ufsm.br; ^4^ Universidade Franciscana, Santa Maria, Rio Grande do Sul, Brazil; ^5^ Graduate Program in Biomedical Gerontology, School of Medicine, Institute of Geriatrics and Gerontology, Pontifícia Universidade Católica do Rio Grande do Sul (PUCRS), Porto Alegre, Rio Grande do Sul, Brazil, pucrs.br; ^6^ Research, Teaching and Technological Development Center, Fundação Universidade Aberta da Terceira Idade, Manaus, Amazonas, Brazil

**Keywords:** mitochondrial dysfunction, oxidative stress, skin aging, therapeutic approach

## Abstract

This narrative review discusses the relationship between structural changes in the skin and mitochondrial function during aging and evaluates emerging therapeutic interventions targeting mitochondrial dysfunction. An analysis of 49 scientific articles published between 2015 and 2025 was conducted using descriptors including “skin aging,” “mitochondrial dysfunction,” “oxidative stress,” and “cutaneous senescence,” and articles were retrieved from PubMed, Scopus, and ScienceDirect. Additional research was conducted using terms related to therapeutic interventions, including “mitochondrial therapies AND skin aging OR cutaneous aging.” Original research articles were included based on thematic relevance, recency, and scientific rigor. The reviewed studies suggest that oxidative stress, mainly from mitochondrial metabolism, is a primary cause of skin cell senescence. Mitochondrial dysfunction emerges as a central mechanistic hub linking oxidative stress, mitochondrial genome instability, chronic low‐grade inflammation (inflammaging), and the senescence‐associated secretory phenotype (SASP) to age‐related structural and functional skin alterations. Mitochondria maintain skin homeostasis through cell proliferation, differentiation, and genetic material synthesis. With advancing age, mitochondrial DNA copy number declines significantly, while reactive oxygen species production increases, thereby compromising cellular energy metabolism. Emerging mitochondrial‐targeted therapeutic strategies, including nicotinamide adenine dinucleotide (NAD^+^) precursors, coenzyme Q10 supplementation, senolytics, and modulators of mitochondrial quality control, show promising effects on skin aging parameters in preclinical and early clinical studies. However, current evidence is based on small clinical trials with short follow‐up periods, and long‐term safety data remain limited. Therefore, while mitochondria are not the sole source of oxidants, growing evidence indicates that oxidative stress‐driven mitochondrial dysfunction represents a priority pathogenic mechanism in skin aging. The clinical translation of mitochondrial‐targeted therapies represents an innovative opportunity for anti‐aging strategies, although the validation of standardized biomarkers and longitudinal safety investigations remains critical for clinical implementation.

## 1. Introduction

Skin aging is a complex biological process with implications that extend beyond esthetics, as it compromises systemic homeostasis and positions dermatological research as a critical pillar of gerontoscience. Among the various theories proposed to explain aging mechanisms, the free radical theory has emerged as one of the most influential. This theory suggests that reactive oxygen species (ROS), generated through both exogenous sources, such as ultraviolet (UV) radiation and environmental pollutants, and endogenous processes during cellular respiration, accumulate over time to cause progressive cellular damage, ultimately contributing to aging at molecular and cellular levels [1, 2].

Mitochondria serve as the primary intracellular source of ROS production, estimated to contribute 1%–5% of total oxygen consumption to the generation of superoxide anions, the predominant ROS species [3]. This mitochondrial ROS production has highlighted the central role of mitochondrial dysfunction in the biological aging process. In the skin, mitochondria play critical roles in maintaining tissue integrity, supporting fundamental cellular processes such as proliferation, differentiation, and apoptosis, as well as in lipid biosynthesis, which is essential for the skin’s barrier function and overall structural integrity [4]. Given these multifaceted roles, a comprehensive understanding of mitochondrial contributions to skin aging is crucial not only for advancing fundamental aging research but also for identifying therapeutic targets to mitigate age‐associated skin decline.

## 2. Methods

This narrative review was conducted with the aim of describing the relationship between structural changes in the skin and mitochondrial function during the aging process. For the preparation of this work, scientific articles published between 2015 and 2025 in English were selected from the databases PubMed, Scopus, and ScienceDirect. The primary search strategy combined controlled terms and free‐text keywords using Boolean operators as follows: (“skin aging” OR “cutaneous aging”) AND (“mitochondrial dysfunction” OR “oxidative stress” OR “cutaneous senescence”). To identify therapeutic approaches, an additional search was conducted using: (“mitochondrial therapy” AND (“skin aging” OR “cutaneous aging”)).

Study selection was based on thematic relevance, recency, and scientific rigor. Original research articles were prioritized. Data extraction and qualitative synthesis were conducted independently by the authors, with discrepancies resolved by consensus. To enhance transparency and methodological quality, reporting followed the SANRA (Scale for the Assessment of Narrative Review Articles) guidance.

To facilitate the understanding of the mechanisms involved, data analysis was organized into thematic sections.

## 3. Results

### 3.1. Structural Alterations in the Skin During Aging: Mechanisms Attributed to Mitochondrial Dysfunction

The skin is a highly specialized organ composed of three main layers—the epidermis, dermis, and hypodermis—each contributing to barrier function, mechanical protection, immune surveillance, thermoregulation, and metabolic homeostasis. The epidermis is primarily formed by keratinocytes organized in stratified layers and is responsible for barrier integrity and renewal capacity. The dermis, rich in fibroblasts, collagen, elastin, and extracellular matrix components, provides tensile strength and elasticity, while the hypodermis contributes to insulation and metabolic support [5]. Mitochondria play a central role in all skin compartments by regulating cellular energy production, redox balance, apoptosis, and biosynthetic pathways essential for tissue maintenance and repair [4]. Due to its complexity, skin aging triggers structural and functional alterations characterized by progressive structural and functional deterioration affecting all layers. Hallmark alterations include epidermal thinning, reduced keratinocyte proliferation, impaired barrier function, dermal atrophy, collagen fragmentation, elastin disorganization, and decreased extracellular matrix synthesis. These changes lead to clinical manifestations such as wrinkles, loss of elasticity, increased fragility, delayed wound healing, and heightened susceptibility to environmental stressors [6].

Accumulating evidence indicates that mitochondrial dysfunction is a key driver of these age‐associated structural changes. Mitochondria are increasingly recognized as key regulators of cellular aging beyond their canonical role in energy metabolism. In aging skin, mitochondrial dysfunction contributes to a complex network of interconnected processes that include oxidative stress, chronic low‐grade inflammation, altered intercellular communication, loss of sensory function, and impaired homeostasis across multiple skin‐resident cell populations [4]. The following are the principal mechanisms through which mitochondria contribute to cellular aging processes.

### 3.2. Oxidative Stress and Mitochondrial Genome Instability

As the primary active site of ROS generation (~1%–5%), particularly superoxide anion, mitochondria play a central role in cellular aging. According to the mitochondrial free radical theory, originally proposed by Denham Harman in 1956, ROS produced endogenously during cellular respiration or generated in response to external agents such as radiation can induce cumulative cellular damage, thereby contributing to the aging process at the molecular level [3, 7].

Oxidative stress damages proteins of the electron transport chain, reducing the efficiency of oxidative phosphorylation and ATP production; this dysfunction enhances free radical generation, establishing a self‐perpetuating cycle within mitochondria. In parallel, mitochondrial DNA (mtDNA)—highly susceptible to ROS due to its proximity to the respiratory chain and the lack of histones—accumulates mutations and deletions that further impair oxidative phosphorylation. This “ROS–mtDNA” axis not only accelerates cutaneous senescence but is also implicated in major non‐communicable chronic diseases, including cancer and type 2 diabetes mellitus, thereby linking skin aging to systemic outcomes of critical relevance for public health [8–10].

Therefore, age‐associated decline in mitochondrial respiratory efficiency leads to electron leakage from the electron transport chain and sustained overproduction of ROS. Excessive mitochondrial ROS not only damages lipids, proteins, and nuclear DNA but also induces cumulative mtDNA mutations and deletions. Given the limited DNA repair capacity of mitochondria, these alterations exacerbate respiratory chain dysfunction, establishing a self‐amplifying cycle of bioenergetic failure and cellular senescence in keratinocytes, fibroblasts, and melanocytes [11].

### 3.3. Mitochondria‐Driven Inflammation and Inflammaging

Beyond oxidative damage, dysfunctional mitochondria actively promote inflammatory signaling in the aging skin. The release of mtDNA, cardiolipin, and other mitochondrial‐derived danger‐associated molecular patterns (DAMPs) into the cytosol or extracellular space triggers innate immune pathways, including the activation of pattern recognition receptors and inflammasome complexes. This process contributes to the establishment of a chronic, low‐grade inflammatory state—commonly referred to as inflammaging—characterized by increased local production of pro‐inflammatory cytokines, chemokines, and matrix‐degrading enzymes. In the dermis, such signaling reinforces extracellular matrix breakdown, while in the epidermis, it disrupts differentiation programs and barrier integrity [12, 13].

### 3.4. Cellular Senescence and the Senescence‐Associated Secretory Phenotype (SASP)

Mitochondrial dysfunction is a critical upstream driver of cellular senescence in skin cells. Senescent keratinocytes and fibroblasts exhibit profound metabolic reprogramming, altered mitochondrial dynamics, and persistent DNA damage responses. These cells acquire a SASP, marked by the secretion of inflammatory mediators, growth factors, and proteases. The accumulation of senescent cells amplifies tissue inflammation, alters stem cell niches, and compromises regenerative capacity, thereby accelerating structural and functional skin aging [14].

### 3.5. PGC‐1α and TFAM in the Regulation of Mitochondrial Biogenesis and Energy Metabolism in Skin Aging

Studies suggest that certain proteins involved in the regulation of mitochondrial biogenesis and the maintenance of cellular energy homeostasis are essential for the functional integrity of the skin [13]. For instance, peroxisome proliferator‐activated receptor gamma coactivator 1‐alpha (PGC‐1α), whose expression is reduced in the aged epidermis, has been shown to play an important role in the regulation of skin regeneration, as it controls the levels of nicotinamide adenine dinucleotide (NAD^+^), which in turn influences the proliferation and fate of epidermal stem cells [15]. Furthermore, PGC‐1α functions as an important transcriptional coactivator that enhances the expression of specific genes through interactions with transcription factors such as Nuclear Respiratory Factor 1 (NRF1) and Nuclear Factor Erythroid 2–Related Factor 2 (NRF2). NRF1 and NRF2 cooperate to promote mitochondrial biogenesis by upregulating mitochondrial transcription factor A (TFAM), another protein considered essential for the maintenance of cutaneous homeostasis, as it acts as a central regulator of mitochondrial gene expression [13].

Consequently, TFAM plays a central role in maintaining mitochondrial function in epidermal cells. TFAM regulates mtDNA transcription and replication and is therefore indispensable for the proper functioning of the electron transport chain. In the epidermis, this protein also participates in the regulation of keratinocyte differentiation, and its deficiency has been associated with disturbances in the energy homeostasis of epidermal cells resulting from impaired mitochondrial biogenesis [16]. In this context, a study conducted by Vidali et al. [17] demonstrated a reduction in TFAM levels in the epidermis of histological sections from elderly individuals compared with those from young donors. This decrease was independent of tissue exposure to UV radiation, suggesting that TFAM deterioration represents an intrinsic feature of cutaneous aging.

### 3.6. Impact of Mitochondria on Epidermal Aging

The epidermis is the outermost layer of the skin and serves as the primary interface between the body and the external environment. It is a highly dynamic, stratified epithelium composed mainly of keratinocytes, along with melanocytes, Langerhans cells, and Merkel cells. Through continuous cell renewal and terminal differentiation, the epidermis forms a resilient barrier that prevents excessive water loss, protects against microbial invasion, and limits exposure to chemical and physical stressors [5].

Beyond its barrier function, the epidermis plays an active role in immune defense, sensory perception, and metabolic regulation. It participates in innate and adaptive immune responses, communicates with cutaneous nerve endings, and supports tissue repair and homeostasis. These functions require substantial metabolic activity and are tightly linked to mitochondrial function. Age‐related alterations in epidermal cellular metabolism and mitochondrial integrity compromise barrier maintenance, immune responsiveness, and neurocutaneous signaling, contributing to the functional decline of aging skin [18].

**Figure 1:**
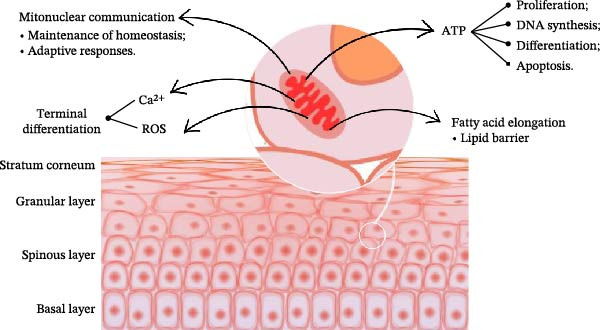
Impact of mitochondrial function on keratinocyte homeostasis. Mitochondria modulate key cellular processes underlying epidermal homeostasis and differentiation through ATP production, thereby influencing proliferation, DNA synthesis, differentiation, and apoptosis. Keratinocyte differentiation is also regulated by reactive oxygen species (ROS) signaling and calcium (Ca^2+^) homeostasis. Mitonuclear communication contributes to the maintenance of cellular homeostasis and adaptive responses. Furthermore, fatty acid elongation is associated with lipid barrier formation. Collectively, these mechanisms are essential for the terminal differentiation of keratinocytes across the epidermal layers (basal, spinous, granular, and stratum corneum). *Source*: Illustration created by the authors using Canva.

Mitochondrial activity exerts a direct influence on epidermal homeostasis and development (Figure 1) through multiple mechanisms: (I) mitochondrial ATP production is indispensable for keratinocyte proliferation, post‐mitotic DNA synthesis, terminal differentiation, and apoptosis, all processes critical for sustaining epithelial homeostasis; (II) ROS are pivotal regulators of keratinocyte terminal differentiation; (III) differentiation of keratinocytes across the epidermal strata depends on the calcium gradient established via mitochondrial uptake of this ion during respiration; and (IV) mitochondrial‐driven fatty acid elongation is essential for the biosynthesis of lipid components that maintain the permeability barrier of the stratum corneum [4].

### 3.7. Impact of Mitochondria on Dermis Aging

The dermis is the connective tissue layer of the skin that provides structural support, elasticity, and mechanical strength. It is composed of a complex extracellular matrix rich in collagen, elastin, and proteoglycans, and contains multiple cell types, including fibroblasts, endothelial cells, immune cells, and nerve fibers. Through this integrated cellular and matrix organization, the dermis ensures tissue resilience, vascular support, sensory function, and effective nutrient and oxygen delivery to the skin. Beyond its structural role, the dermis actively regulates wound healing, immune surveillance, and intercellular signaling. These functions rely on continuous extracellular matrix remodeling, vascular homeostasis, and tightly regulated inflammatory responses, all of which are energetically demanding and closely linked to mitochondrial function. Fibroblasts are the principal mesenchymal cells of the dermis and play a central role in maintaining skin structure and mechanical integrity. They are responsible for the synthesis, organization, and remodeling of the extracellular matrix, including collagen, elastin, and proteoglycans, which provide tensile strength, elasticity, and resilience to the skin. Through continuous matrix turnover, fibroblasts support tissue stability and enable effective responses to mechanical stress and injury [19].

Mitochondria also play a pivotal role in the physiology of dermal fibroblasts, which are highly energy‐dependent for extracellular matrix maintenance. Mitochondrial dysfunction in fibroblasts impairs the synthesis of collagen and other matrix components, reduces proliferative capacity, and induces morphological changes associated with the accumulation of defective organelles. Moreover, it promotes the activation of matrix metalloproteinases, enzymes responsible for collagen fiber degradation. Collectively, these alterations are closely linked to the accumulation of mtDNA mutations, reduced ATP production, defective mitophagy, imbalance in mitochondrial dynamics, and increased production of ROS and inflammatory cytokines, ultimately driving the establishment of the SASP [12].

### 3.8. Intrinsic and Extrinsic Skin Aging Components

The cutaneous aging process is highly associated with intrinsic and extrinsic components [20, 21]. Intrinsic aging, also referred to as chronological aging, is primarily governed by genetically programmed processes and the passage of time. It is characterized by progressive cellular senescence, reduced proliferative capacity of skin cells, telomere shortening, epigenetic alterations, and cumulative mitochondrial dysfunction. These mechanisms lead to decreased collagen synthesis, epidermal thinning, impaired barrier function, reduced vascularization, and diminished regenerative potential [21].

In the intrinsic component, in addition to redox imbalance, there is a loss of mitochondrial membrane potential, mtDNA mutations, reduced mitophagy, and fragmentation of the mitochondrial network, all of which promote the gradual decline of mitochondrial function. Furthermore, the reduction of key molecules such as Coenzyme Q10 (CoQ10), responsible for electron transport between mitochondrial complex I/II and complex III, compromises both energy production and antioxidant defense. Collectively, these factors lead to decreased energy production, increased oxidative stress, and impaired cellular renewal, resulting in skin thinning, loss of elasticity, and the formation of fine wrinkles—hallmarks of chronological aging [22–24].

Extrinsic skin aging is primarily driven by environmental and lifestyle‐related factors that accelerate cellular and molecular damage beyond intrinsic aging mechanisms, with mitochondrial dysfunction emerging as a central mediator of these effects. Chronic UV radiation exposure is the most significant extrinsic factor, inducing mtDNA damage, impairing oxidative phosphorylation, and increasing ROS production. Air pollution, including particulate matter and ozone, further exacerbates mitochondrial oxidative stress and disrupts redox homeostasis in skin cells. Smoking introduces toxins that directly inhibit mitochondrial respiratory enzymes and promote persistent inflammatory signaling. Additional contributors, such as poor nutrition, psychosocial stress, sleep disruption, and repetitive mechanical stress, interfere with mitochondrial biogenesis, energy metabolism, and cellular repair processes [25].

Collectively, these extrinsic components converge on mitochondrial dysfunction, amplifying oxidative damage, inflammation, and premature cellular senescence, thereby leading to loss of firmness and increased skin fragility [25]. Especially, photoaging exacerbates these processes through UV radiation, which induces direct mtDNA damage and a persistent inflammatory response. This combination accelerates extracellular matrix degradation, promotes irregular hyperpigmentation and deep wrinkles, and is associated with an increased risk of cutaneous carcinogenesis [22, 26].

### 3.9. Protective and Adaptive Mitochondrial Responses

Given the central role of mitochondria in coordinating numerous cellular functions and their vulnerability to oxidative stress‐induced damage—which can impair mitochondrial function and potentially trigger cell death—it becomes essential to maintain quality control mechanisms capable of preserving mitochondrial integrity and safeguarding tissues from the detrimental effects of dysfunctional organelles [27].

Evidence suggests that mitochondria possess more robust protective mechanisms than previously believed. As reported by Jiao et al. [28], one such mechanism is mitocytosis, in which dysfunctional mitochondria are selectively transported out of the cell and subsequently discarded via structures termed migrasomes by the authors. Although this phenomenon is still recent and remains poorly explored, it represents a potential pathway for maintaining mitochondrial homeostasis, the in vivo relevance of which still requires confirmation.

Another fundamental mechanism involved in maintaining mitochondrial homeostasis is mitophagy. This process refers to the selective removal of damaged or dysfunctional mitochondria from cells, thereby preserving mitochondrial quality and cellular function. The PINK1/Parkin pathway is a well‐established mediator of mitophagy, in which PINK1 accumulates on the outer mitochondrial membrane of damaged mitochondria, recruiting and activating Parkin. Activated Parkin builds polyubiquitin chains on proteins located on the mitochondrial membrane, thereby “marking” the organelle for degradation and promoting the recruitment of proteins associated with the autophagosomal membrane. The autophagosomal membrane subsequently engulfs the damaged mitochondrion, forming a mitophagosome that later fuses with lysosomes to generate an autophagolysosome, where the mitochondrion is degraded [29].

In this context, in vitro [30, 31] studies have sought strategies capable of attenuating mitochondrial dysfunction associated with photoaging through the induction of mitophagy by stimulating the PINK1/Parkin signaling pathway.

Mitophagy is one of the mechanisms through which mtDNA integrity is preserved. In addition, other processes, including the organization of mtDNA into nucleoids and the balanced dynamics of mitochondrial fission and fusion, also contribute to the maintenance of mitochondrial homeostasis. Furthermore, studies indicate that aging‐related phenotypes become manifest only when the mutational load in mtDNA exceeds critical thresholds (60%–90%) [7].

**Figure 2:**
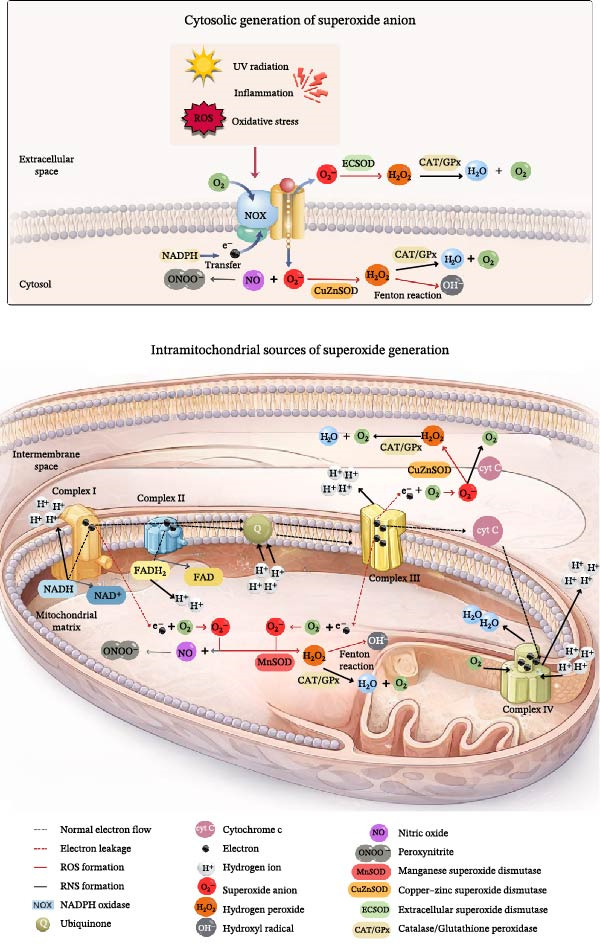
Mechanisms of superoxide (O_2_^−^) anion generation in mitochondria and cytosol. Sources and biochemical pathways of superoxide generation in the cytosol and mitochondria. Cytosolic superoxide production can occur through the activity of NADPH oxidase (NOX), stimulated by factors such as UV radiation, inflammation, and oxidative stress. The superoxide anion (O_2_•^−^) can react with nitric oxide (NO) to form peroxynitrite (ONOO^−^) or be converted into hydrogen peroxide (H_2_O_2_) by superoxide dismutases (SOD), including CuZnSOD in the cytosol and EC‐SOD in the extracellular space. H_2_O_2_ is further detoxified into water (H_2_O) and oxygen (O_2_) by catalase (CAT) and glutathione peroxidase (GPx), although it may also generate hydroxyl radicals (•OH) via the Fenton reaction. In mitochondria, electron leakage from the electron transport chain—mainly at complexes I and III—results in superoxide formation within the mitochondrial matrix and intermembrane space. Mitochondrial superoxide is converted to H_2_O_2_ by manganese superoxide dismutase (MnSOD), contributing to the balance between reactive oxygen species (ROS) production and antioxidant defense mechanisms. *Source:* Illustration created by the authors using Canva.

Regarding ROS generation, it is important to acknowledge their dual role: at physiological levels, they act as signaling molecules, whereas excess promotes oxidative stress. This imbalance arises when the production of ROS and reactive nitrogen species exceeds the cell’s antioxidant capacity. Within mitochondria, the superoxide anion is the primary species formed and, if not neutralized by superoxide dismutase, can give rise to more potent oxidants such as the hydroxyl radical and peroxynitrite (Figure 2) [32]. In the skin, this process is particularly relevant, as keratinocytes and fibroblasts exhibit an age‐related decline in antioxidant defenses, favoring the onset of cellular senescence and photoaging [33].

A key point to highlight is the role of mitochondria as the primary intracellular source of superoxide anion, particularly under conditions of respiratory chain dysfunction. In this context, superoxide dismutase 2 (MnSOD or SOD2), located in the mitochondrial matrix, constitutes one of the major endogenous systems for the detoxification of the superoxide anion, playing a crucial role in protection against oxidative damage. Owing to its strategic localization and antioxidant function, SOD2 is essential for the maintenance of mitochondrial redox homeostasis and for the survival of aerobic organisms [34].

The deepening of knowledge regarding mitochondrial adaptive and protective mechanisms paves the way for therapeutic strategies aimed not only at reducing oxidative stress but also at enhancing endogenous mitochondrial quality control mechanisms, thereby offering new perspectives for the modulation of the aging process [35].

### 3.10. Therapeutic Implications and Clinical Translation

The growing understanding of mitochondrial dysfunction as a central mechanism of skin aging has opened new therapeutic possibilities. As mitochondria deteriorate with age, their capacity for energy production declines while ROS generation increases, triggering a cascade of cellular damage that manifests as visible and structural signs of skin aging. This knowledge has driven not only the identification of antioxidant compounds but also the development of pharmacological and metabolic interventions aimed at preserving mitochondrial function and enhancing endogenous defense systems [36].

Antioxidants represent a diverse group of compounds, including vitamins (C and E), minerals (such as selenium and zinc), plant‐derived bioactive compounds (flavonoids and polyphenols), and synthetic substances such as N‐acetylcysteine. In general, their primary mechanism involves the neutralization of ROS, thereby preventing oxidative damage to lipids, proteins, and DNA [37].

In addition to direct neutralization, antioxidants act by regenerating endogenous antioxidant systems, chelating transition metals involved in ROS generation, and modulating intracellular signaling pathways. The latter mechanism involves the activation of transcription factors such as NRF2, which positively regulates the expression of endogenous antioxidant enzymes, thereby enhancing the cell’s capacity to withstand oxidative stress and maintaining cellular redox balance [38].

For example, N‐acetylcysteine, an acetylated cysteine residue, primarily acts as an indirect antioxidant due to its ability to promote the synthesis of glutathione, a key antioxidant enzyme. This occurs through the release of cysteine, which, in addition to serving as an important precursor for glutathione synthesis, also acts directly as an effective free radical scavenger, protecting against photoaging [39]. Although no studies have investigated its role in mitochondrial dysfunction in skin cells, in the human monocytic cell line THP‐1, pre‐treatment with N‐acetylcysteine attenuated rotenone‐induced damage to mitochondrial complex I, reducing the release of mtDNA and the NDUFS7 subunit, thereby demonstrating structural protection and preservation of mitochondrial function [40].

Building on the antioxidant approach, researchers have identified specific compounds that demonstrate efficacy in protecting mitochondrial function and combating skin aging. The therapeutic impact of salvianolic acid B, a compound isolated from *Salvia miltiorrhiza* with high antioxidant capacity, was evaluated against UVB‐induced skin photoaging. Sun et al. [41] conducted in vitro and in vivo experiments and demonstrated that salvianolic acid B aids in mitochondrial protection through the suppression of ROS production and activation of the transcription factor NRF2. The authors concluded that salvianolic acid B represents an effective antiphotoaging therapy, even surpassing the effects of tretinoin (Retinol‐A) in reducing epidermal thickness, increasing dermal collagen, and decreasing the expression of senescence‐associated genes following UVB irradiation [42].

Another promising compound in this therapeutic approach is CoQ10. This lipophilic molecule is essential for mitochondrial electron transport; in its reduced form, it stabilizes membranes, prevents lipid peroxidation, regenerates vitamins C and E, and modulates pathways such as MAPK, thereby reducing matrix metalloproteinases and collagen degradation, with a direct impact on the dermal extracellular matrix [43–45]. In a randomized clinical trial involving 73 healthy volunteers, topical CoQ10 formulations reduced acute UV‐ and stress‐induced damage and promoted long‐lasting anti‐aging effects on the skin, supporting its translational relevance in skincare [42].

There is also a class of antioxidants specifically designed to act directly within mitochondria, referred to as mitochondria‐targeted antioxidants. This therapeutic strategy has emerged as a promising approach to mitigate mitochondrial dysfunction and oxidative stress. Unlike conventional antioxidants, which generally distribute throughout different cellular compartments, mitochondria‐targeted antioxidants preferentially accumulate within mitochondria, where they can directly neutralize ROS, limit oxidative damage to mtDNA, and contribute to the preservation of mitochondrial bioenergetics [46].

Among the most extensively studied mitochondria‐targeted antioxidants are MitoQ (ubiquinone conjugated to triphenylphosphonium) and SkQ1 (plastoquinone conjugated to triphenylphosphonium). In MitoQ, the ubiquinone moiety corresponds to the oxidized form of CoQ10 and is inherently lipophilic, exhibiting limited aqueous solubility and therefore lacking efficient mitochondrial targeting when administered in its native form. Similarly, SkQ1 contains a plastoquinone moiety, an electron carrier naturally present in chloroplasts and cyanobacteria, which can be reduced to plastoquinol, its antioxidant‐active form. Due to the lipophilic nature of these quinones, efficient mitochondrial accumulation requires a carrier. Conjugation to triphenylphosphonium, a lipophilic cation, enables selective mitochondrial uptake driven by the negative membrane potential of the inner mitochondrial membrane. Experimental studies indicate that, once accumulated within mitochondria, these compounds can attenuate mitochondrial oxidative damage, reduce inflammatory signaling, and mitigate cellular senescence, suggesting potential therapeutic benefits in conditions associated with mitochondrial dysfunction [46–48].

A complementary approach to antioxidant strategies is the modulation of mitochondrial turnover through specific pharmacological agents. Rapamycin exemplifies this strategy by acting as an inhibitor of the mTOR (mechanistic target of rapamycin) signaling cascade, a kinase that functions through two distinct complexes—mTORC1 and mTORC2—each exhibiting varying degrees of drug responsiveness. When mTORC1 activity is inhibited, autophagic mechanisms are activated, particularly mitophagy. Although still exploratory, human studies suggest that topical rapamycin reduces markers of skin senescence, highlighting its translational potential [49].

A third promising therapeutic avenue focuses on addressing the age‐related decline in cellular NAD + levels, a critical cofactor for mitochondrial function and cellular repair mechanisms. NAD + plays fundamental roles as a cofactor in multiple metabolic pathways, including energy metabolism, fatty acid β‐oxidation, and the tricarboxylic acid cycle. Moreover, it serves as an essential substrate for enzymes critical to the aging process, particularly sirtuins and poly(ADP‐ribose) polymerases, which coordinate cellular processes such as DNA repair, chromatin epigenetic modifications, and the regulation of cellular senescence [36].

Age‐related declines in NAD + levels compromise bioenergetic pathways and key enzymes (sirtuins and PARPs), affecting DNA repair, epigenetic regulation, and senescence control. Repletion strategies—topical or systemic—restore sirtuin activity, stimulate autophagy, and improve mitochondrial functionality, representing a promising approach against skin aging [36, 50].

## 4. Conclusion

It is undeniable that mitochondria play complex roles in skin aging, given their importance for the physiological functions of keratinocytes and fibroblasts. Accordingly, skin aging should be understood as a multifactorial process, in which mitochondrial dysfunction, integrating oxidative stress, cellular senescence, and declines in energy metabolism, represents a key element but interacts with other mechanisms, such as nuclear damage, chronic inflammation, and deficiencies in antioxidant systems.

Recent evidence suggests that mitochondria‐targeted strategies—such as NAD + precursors, CoQ10, mitochondria‐targeted antioxidants, senolytics, and autophagy modulators—show promising results in improving skin parameters and delaying age‐associated functional decline. However, these findings are largely based on early‐stage clinical studies with small sample sizes, short follow‐up periods, and limited demographic diversity, which constrains the generalizability of the results.

For these approaches to be incorporated into dermatological protocols, longitudinal, multicenter investigations with greater methodological standardization are required, including the validation of specific biomarkers of mitochondrial function in the skin. In summary, advances in understanding the interface between mitochondrial biology and skin aging open new therapeutic perspectives, with the potential to transform skin health maintenance in aging populations.

## Author Contributions

Made substantial contributions to conception and design of the study: Nathália Cardoso de Afonso Bonotto, Fernanda Barbisan, Ivana Beatrice Mânica da Cruz, and Elize Musachio. Performed data analysis and interpretation: Nathália Cardoso de Afonso Bonotto and Fernanda Barbisan. Performed data acquisition: Nathália Cardoso de Afonso Bonotto and Fernanda Barbisan. Investigation, resources: Giulliano Danezi Felin and Giancarllo Danezi Felin. Provided technical support: Elize Musachio, Carla Helena Augustin Schwanke, and Ivana Beatrice Mânica da Cruz.

## Funding

This work was supported by the Scholarship Number 88887.715145/2022‐00 from the Brazilian funding agency Coordination for the Improvement of Higher Education Personnel (CAPES).

## Conflicts of Interest

The authors declare no conflicts of interest.

## Data Availability

The data that support the findings of this study are available from the corresponding author upon reasonable request.
